# A randomized controlled double-blind study of rotigotine on neuropsychiatric symptoms in de novo PD

**DOI:** 10.1038/s41531-020-00142-x

**Published:** 2020-12-15

**Authors:** A. Castrioto, S. Thobois, M. Anheim, J. L. Quesada, E. Lhommée, H. Klinger, A. Bichon, E. Schmitt, F. Durif, J. P. Azulay, J. L. Houeto, N. Longato, C. Philipps, P. Pelissier, E. Broussolle, E. Moro, C. Tranchant, V. Fraix, P. Krack, for the Honeymoon study group

**Affiliations:** 1 Movement disorders Center, Neurology, CHU Grenoble Alpes, Grenoble, France; 2 Univ. Grenoble Alpes, Inserm, U1216, CHU Grenoble Alpes, Grenoble Institute Neurosciences, 38000 Grenoble, France; 3 Univ Lyon, Université Claude Bernard Lyon 1, CNRS, Institut des Sciences Cognitives, Bron, France; 4 Hospices Civils de Lyon, Hôpital Neurologique Pierre Wertheimer, Neurologie C, Bron, France; 5 Département de neurologie, Hôpital de Hautepierre, Hôpitaux Universitaires de Strasbourg, Strasbourg, France; 6 Institut de Génétique et de Biologie Moléculaire et Cellulaire, (IGBMC), INSERM-U964/CNRS-UMR7104/, Université de Strasbourg, Illkirch, France; 7 Fédération de Médecine Translationnelle de Strasbourg (FMTS), Université de Strasbourg, Strasbourg, France; 8 Clinical Pharmacology Unit, INSERM CIC 1406, CHU Grenoble Alpes, Grenoble, France; 9 Université Clermont Auvergne, EA7280 NPsy-Sydo, Clermont-Ferrand University hospital, Neurology Department, Clermont-Ferrand, France; 10 Neurology and Pathology Department of the Movement, University Hospital of Marseille, Timone Hospital, 264, rue Saint-Pierre, 13385 Marseille, France; 11 Service de Neurologie, CIC-INSERM 1402, CHU de Poitiers, Université de Poitiers, Poitiers, France; 12 Department of Neurology, Inselspital, University Hospital Bern, CH-3010 Bern, Switzerland

**Keywords:** Parkinson's disease, Parkinson's disease, Human behaviour, Anxiety, Depression

## Abstract

Management of apathy, depression and anxiety in Parkinson’s disease (PD) represents a challenge. Dopamine agonists have been suggested to be effective. This multicenter, randomized (1:1), double-blind study assessed the 6-month effect of rotigotine versus placebo on apathy, depression and anxiety in de novo PD. The primary outcome was the change of apathy, measured with the LARS. The secondary outcomes were the change in depression and anxiety, measured with BDI-2 and STAI-trait and state. Forty-eight drug-naive PD patients were included. The primary outcome was not reached, with a surprisingly high placebo effect on apathy (60%). There was no significant difference in the change of depression at 6 months between rotigotine and placebo. Trait-anxiety was significantly improved by rotigotine compared to placebo (*p* = 0.04). Compared to placebo, low dose rotigotine significantly improved trait anxiety, but not apathy and depression. The major placebo effect on apathy points towards the importance of a multidisciplinary and tight follow-up in the management of neuropsychiatric symptoms.

## Introduction

Neuropsychiatric symptoms are common and disabling in Parkinson’s disease (PD)^1,2^.

Apathy, a disorder characterized by lack of motivation and goal-directed behaviors, affects almost 40% of PD patients in isolation, or with depression and anxiety^2–4^. In untreated PD patients, apathy ranges from 19 up to 33%^5–7^. The pathophysiology of apathy is still under investigation. Several studies suggest a prominent dopaminergic deficit in the mesolimbic system, as a main contributor^8–14^. The dopaminergic medication has been shown to improve motivation and apathy in PD patients^8–14^. A serotonergic system dysfunction has been advocated more recently^15,16^. Last, but not least, the cholinergic system might play an important role in the pathogenesis of apathy especially in association with cognitive impairment, as shown by its improvement with acetyl-cholinesterase inhibitors^17^. Untreated PD rarely present cognitive impairment^18^ and as such the role of the cholinergic system to apathy might be less relevant at this stage.

Depression and anxiety are also very common in PD and seem in part related to the dopaminergic deficit, as suggested by their improvement with dopaminergic treatments^2,19^.

Apathy, depression and anxiety have been conceptualized as hypodopaminergic symptoms related to PD, and opposed to hyperdopaminergic behaviors, including impulse control disorders, related to dopaminergic treatment^20^.

Studying apathy in de novo PD patients might be contributive, since they represent a homogeneous population, without the confounding effect of antiparkinsonian drugs. Unfortunately, studies in de novo PD patients are scarce and mostly lack thorough neuropsychological evaluation. To our knowledge, there are no randomized-controlled studies specifically designed to assess the efficacy of a dopamine agonist on apathy and hypodopaminergic behaviors in de novo PD.

The aim of this randomized-controlled study was to assess the effect of rotigotine, a 24-h continuous transdermal non-ergot dopamine agonist^21^ on apathy, depression and anxiety in de novo PD.

## Results

**Fig. 1:**
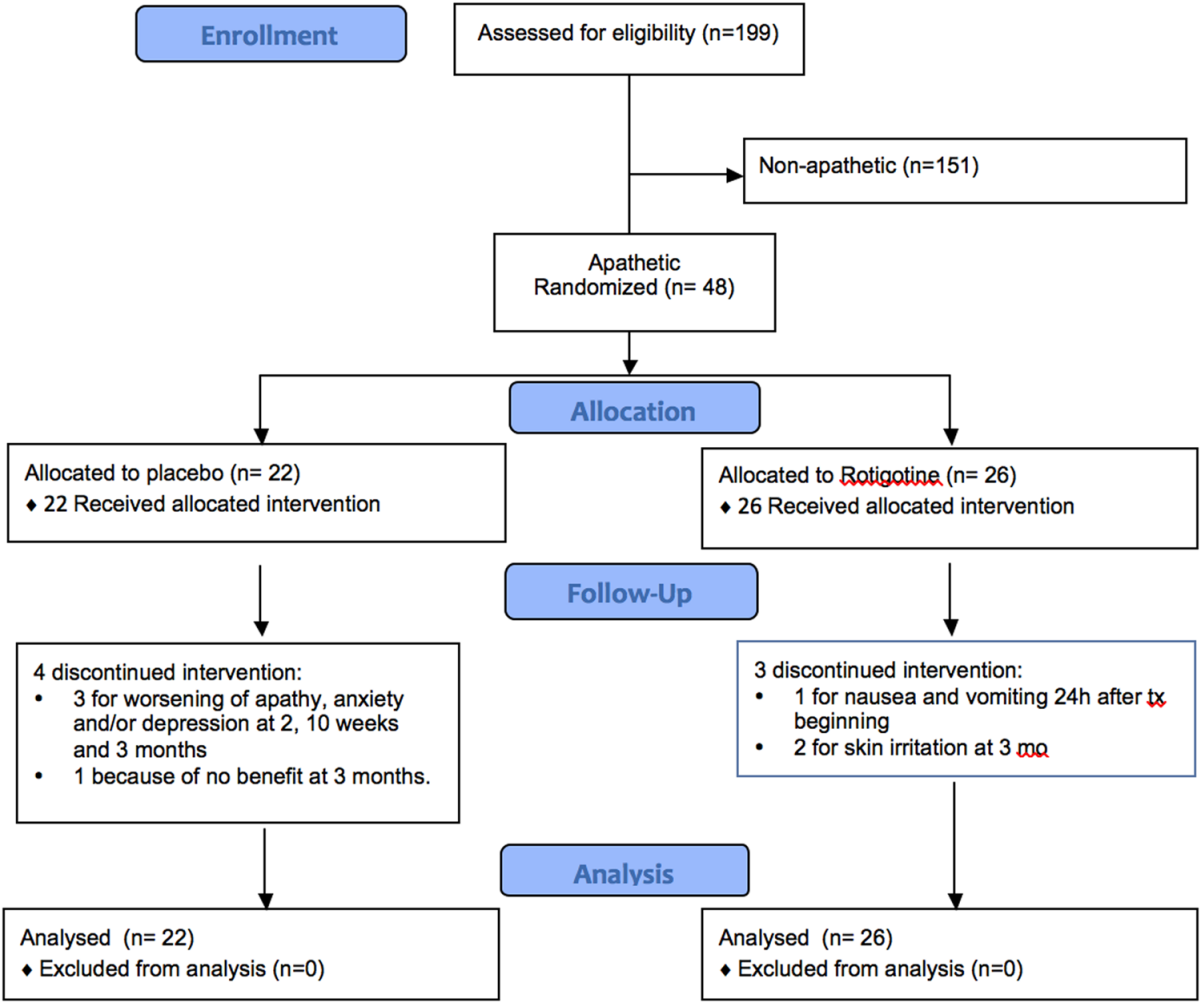
Consort flow diagram. Screening of de novo PD patients for apathy, randomization to rotigotine or placebo, follow-up and analysis.

We included 48-PD patients. Twenty-two patients were allocated to placebo and 26 to active treatment (Fig. 1). Baseline characteristics of patients are listed in Table 1.

**Table 1:** Baseline characteristics of patients.

Baseline	48 patients	22 under placebo	26 under rotigotine
Age	58.8 ± 7.6 (44.6; 70.2)	60.9 ± 8.4 (44.6; 70.2)	57.1 ± 6.5 (45.1; 67.9)
Disease duration (mo)	25 ± 17.1 (0; 80)	24 ± 18 (3; 61)	26 ± 16.4 (0; 80)
Sex (male)	32 (66.7%)	15 (68.2%)	17 (65.4%)
FAB	16.7 ± 1 (15; 18)	16.8 ± 1.1 (15; 18)	16.6 ± 1.0 (15; 18)
MDRS	139.9 ± 2.9 (133; 144)	139.6 ± 3.1 (133; 144)	140.2 ± 2.8(133; 144)
Rasagiline (No. of pts.)	14	5	9
Antidepressant (No. of pts.)	10	5	5

Mean ± SD (min; max).

*FAB* Frontal assessment battery, *MDRS* Mattis Dementia Rating scale.

7 patients discontinued the study prematurely. Among patients under rotigotine, there were one drop off 24 h after beginning the treatment, because of nausea and vomiting, and two at 3 months because of skin irritation. Among patients under placebo, three discontinued the study because of worsening of apathy, anxiety and/or depression (at 2, 10 weeks and 3 months), and one because of no benefit at 3 months.

The mean dose reached at the end of the titration phase in the placebo group was 8 ± 0 mg/day, and in the rotigotine group 7 ± 1.8 mg/day.

At baseline, 27/48 patients had concomitant anxiety symptoms, 19/48 had concomitant depressive symptoms, and 16/48 had both anxiety and depression symptoms (eight in the placebo group and eight in the rotigotine group), as measured with a score of the dedicated item of the Ardouin scale of behavior in PD ≥ 2 (Table 2).

**Table 2:** Number of apathetic patients with anxiety state, depression state or both measured with a score of the dedicated item of the Ardouin scale of behavior in PD ≥ 2.

	Placebo		Rotigotine	
	Baseline	6 months	Baseline	6 months
Anxiety	15	12	12	5
Depression	9	10	10	5
Anxiety and depression	8	7	8	4

### Main outcome

Both rotigotine and placebo reduced Lille Apathy rating scale (LARS) scores at 6 months compared to baseline (Table 3, mean difference between 6-month and baseline LARS scores for rotigotine group −8 ± 10, −58.4%; for placebo group −6.6 ± 8.3, −60%). There was no statistical difference between rotigotine and placebo group (delta 1.3 ± 2.7, *p* = 0.63). Results remain non-significant also when adjusting analyses on baseline depression or anxiety scores.

**Table 3:** Neuropsychiatric, motor and quality of life scores at baseline and at 6 months.

	Placebo		Rotigotine		*F*	*P*
	Baseline	6 months	Baseline	6 months		
Apathy (LARS)	−11 ± 6.9 (−21; 2)	−17.6 ± 9.5 (−34; 1)	−13.7 ± 6.9 (−21; 4)	−21.7 ± 8.8 (−33; −2)	0.24	0.6268
Depression (BDI-2)	18.1 ± 6.1 (5; 29)	14.5 ± 7.4 (3; 26)	17 ± 8.7 (4; 35)	12.2 ± 8.9 (2; 35)	0.29	0.5945
Anxiety (STAI-state)	41.5 ± 11.3 (21; 61)	39.1 ± 10.4 (20; 55)	38.7 ± 11.2 (23; 70)	36 ± 12 (20; 67)	0.01	0.9360
Anxiety (STAI-trait)	51.8 ± 9.4 (33; 66)	50.5 ± 9.5 (30; 71)	50.2 ± 8.7 (32; 64)	43.5 ± 10.9 (26; 64)	4.45	0.0405
Apathy (Starkstein apathy scale)	20.5 ± 4.8 (11; 29)	18.8 ± 6.4 (6; 29)	18.6 ± 3.7 (13; 28)	14 ± 5.8 (5; 23)	2.57	0.1156
Apathy (Ardouin)	2.41 ± 0.67 (1; 4)	1.76 ± 1.09 (0; 4)	2.15 ± 0.67 (1; 4)	1 ± 0.94 (0; 3)	2.82	0.1002
Depression (Ardouin)	1.36 ± 0.95 (0; 3)	1.27 ± 1.08 (0; 3)	1.31 ± 0.93 (0; 3)	0.77 ± 0.86 (0; 3)	1.82	0.1837
Anxiety (Ardouin)	1.68 ± 1.13 (0; 4)	1.36 ± 1 (0; 3)	1.38 ± 0.94 (0; 3)	0.85 ± 0.92 (0; 3)	0.71	0.4024
Hyperdopaminergic behaviors (Ardouin)	0.95 ± 1.29 (0; 4)	1.27 ± 1.32 (0; 4)	0.72 ± 1.28 (0; 5)	2.44 ± 2.27 (0; 7)	6.82	0.0122
MDS-UPDRS III	25.1 ± 9.4 (8; 48)	28.4 ± 12 (11; 54)	27.8 ± 10.8 (12; 57.5)	25.9 ± 11.6 (4; 47)	6.33	0.0154
PDQ-39	5.7 ± 2.4 (2; 12.3)	5.3 ± 2.1 (2.1; 10)	5.9 ± 2.6 (2.3; 11.6)	6.0 ± 2.5 (2; 11.6)	1.18	0.2825

Mean ± SD (min; max).

### Secondary outcomes

There was no significant difference in the change of depression, measured with the BDI-2 scores between placebo (−3.6 ± 8.2, −19.9%) and rotigotine (−4.7 ± 6.5, −27.6%).

There was a statistically significant difference in the change of anxiety measured with the STAI-trait, adjusted on the initial level of the parameter (*p* = 0.04), with a reduction of anxiety in the rotigotine group of 6.7 ± 8.4 points (13.3%) and of 1.3 ± 9.4 points (2.5%) in the placebo group.

The change of anxiety measured with the STAI-state score, adjusted on the initial level of the parameter (−2.4 ± 10.2, −5.8% for the placebo group, −2.6 ± 10.7, −6.8% for the rotigotine group) was not significant.

### Exploratory outcomes

Beyond the LARS, other dedicated scales were used to assess apathy.

When assessed with the Starkstein apathy scale, apathy decreased at 6 months in the rotigotine group (−4.58 ± 5.62, 24.6%), although the difference with group placebo (−1.73 ± 6.7, 8.4%) was not significant (*p* = 0.12).

The Ardouin Scale of Behavior in PD showed a trend toward a reduction in apathy in the rotigotine group at 6 months although not significant (−1.15 ± 0.97, −53.6% under rotigotine, −0.67 ± 1.02, −27.7% under placebo, *p* = 0.1).

Concerning the depression item of the Ardouin Scale of Behavior in PD, there was no statistical difference between the two groups in the change over time (*p* = 0.18), although there was a trend toward reduction of depression score only under rotigotine (−0.54 ± 1.03, −41.2% under rotigotine, −0.09 ± 1.27, −6.7% under placebo).

Similarly, assessing anxiety with the Ardouin Scale of Behavior in PD, there was a trend toward reduction in anxiety at 6 months in the rotigotine group, although there was no statistically difference between the two groups over time (−0.54 ± 0.95, −38.9% under rotigotine, −0.32 ± 0.84, −18.9% under placebo, *p* = 0.4).

Moreover, there was a significant difference between the two groups in the change at 6 months of the hyperdopaminergic behaviors assessed with the Ardouin Scale of Behavior in PD, with an increase in the rotigotine group (1.72 ± 2.28, 239% under rotigotine, 0.32 ± 1.13, 33.3% under placebo, *p* = 0.01).

The number of patients presenting with comorbidity of apathy, anxious and/or depressive symptoms (measured with a score ≥2 of the specific item of the Ardouin Scale of Behavior in PD) halved at 6 months in the group under rotigotine, whereas it did not change in patients under placebo (Table 2).

Concerning motor symptoms, there was a statistical significant difference between groups in the change of the severity of motor symptoms measured by the MDS-UPDRS motor score at 6 months with an improvement in the rotigotine group (*p* = 0.02) (Table 3).

There was no statistical significant difference between groups in the change of the PDQ39 score between baseline and 6 months (Table 3), adjusted on the initial level of the parameter (0.1 ± 1.7, 2% under rotigotine, −0.42 ± 1.59, −7% under placebo, *p* = 0.28).

### Adverse events

Thirty-five adverse events (16 considered as unrelated to treatment) were reported (16 in the placebo group and 19 in the rotigotine group). A worsening of behavioral hypodopaminergic syndrome was reported in five patients under placebo, but in none under rotigotine. Three serious adverse events were reported in the rotigotine group (a vasovagal episode, sciatica, and cruralgia), two in the placebo group (worsening of hypodopaminergic syndrome).

## Discussion

The present study represents the first randomized-controlled study assessing rotigotine effects on apathy and other neuropsychiatric symptoms with a thorough neuropsychological evaluation in de novo PD patients. In this randomized-controlled trial, a marked improvement of apathy in de novo PD patients was found under both placebo (60%) and rotigotine (58.4%). The between groups difference in the change of apathy, as measured with the LARS at 6 months, was not statistically significant and, as such, the primary outcome was not reached. Furthermore, trait anxiety, a persisting and enduring anxiety status that could predispose persons to state anxiety at times of stress^22^, significantly improved only in the rotigotine group. However, this result should be taken cautiously since state anxiety, i.e., a temporary anxiety at the precise moment of evaluation, did not significantly change with rotigotine. Lower baseline state anxiety scores might partly explain the lack of significance.

Treatment with dopamine agonist can induce hyperdopaminergic behaviors^20,23^. Therefore, it is not surprising that rotigotine in our study led to an increase in hyperdopaminergic behaviors. None of the patients, however, developed impulse control disorders, or punding, or psychosis. Mild, non-pathologic hyperdopaminergic symptoms, measured with the Ardouin scale, such as an increase in hobbyism, positively correlate with quality of life^24^. These findings point towards a reduction of apathy, depression, and anxiety symptoms and an increase of hyperdopaminergic behavior in PD patients treated with rotigotine^2,25^.

Several studies have shown rotigotine efficacy on non-motor symptoms^13,26–29^, including mood and apathy. However, these studies concerned more advanced PD patients and, were not specifically designed to assess apathy and neuropsychiatric symptoms. Rasagiline, and levodopa have also been shown to improve apathy^2^. In advanced PD patients treated with subthalamic stimulation, postoperative apathy has been shown to be improved by piribedil, a dopamine agonist, in a randomized-controlled study^11^. One reason for a not significant effect in our study may be first related to the use of relatively low doses of rotigotine, as recommended in early stages of the disease (up to 8 mg/day), much lower to the equivalent ones used in the successful study using piribedil^11^. Importantly, equivalences among agonists and L-dopa are calculated based on motor effects, but no data are available about their equivalent dose based on psychotropic effects, an obvious limitation when targeting the hypodopaminergic psychological triad.

The lack of efficacy may also derive from methodological issues. Indeed, despite the negative primary outcome, our study points to potential benefits of dopamine agonists on apathy using other outcome measures demonstrating the importance of the neuropsychological tools used. When using the Starkstein apathy scale and the Ardouin scale, rotigotine reduced apathy with a smaller placebo effect (8% and 28% respectively) than with the LARS scale. This underlines that the choice of a scale has important implications, since different scales have different purposes and psychometric properties^30,31^ and thus can lead to different results. Furthermore, the same apathy scale can be scored differently when rated by the patient or the caregiver^32^. Patients may have difficulties judging their own motivation. There is a lack of data concerning the convergent and discriminant validity of different apathy scales, leading to a lack of a well-accepted “gold standard” for the assessment of apathy^31^. This can explain such different results with different tool concerning the same symptom in our study. The LARS scale is a structured interview, including 33 items, and has been validated in PD^33^. The Starkstein apathy scale consists of 14 questions, to be answered by the patient^34^. Compared to the LARS, the questions are less specific. The Ardouin scale^35^ probably is a good compromise between the two scales, since it is based on clinical impression, by the way of a semi-structured interview and may be a more reliable tool of assessment of behavior than an autoevaluation.

Furthermore, patients under placebo had a large improvement of apathy. This might be explained by the well-known placebo-induced dopamine release in the mesolimbic projection to the ventral striatum, which could specifically act on motivation^36^. Depression also seems highly sensitive to the placebo effect. In major depression, it has been shown that the placebo effect is higher in mild than in severe depression and that it is easier to demonstrate a beneficial effect of antidepressants in severe than in mild depression^37^. The mild severity of depression in this study might contribute to a higher placebo effect. On the opposite motor symptoms, depending on dopaminergic denervation of the dorsal striatum, showed no placebo effect in this study.

Moreover, beyond the placebo effect, the very tight neurological and psychological follow-up and the thorough neuropsychological assessment provided here represent an uncommon level of care that, besides any drug intake, may explain the major improvement of neuropsychiatric disorders. This stands particularly true in the delicate phase, which follows the diagnosis announcement of a chronic neurological disease. Therefore being followed-up in a referral center, with a dedicated time for expressing the distress, might have contributed, per se, to the improvement of apathy, and other neuropsychological symptoms in our study, making, a posteriori, very unlikely the possibility of observing a significant add-on impact of rotigotine. In the same vein, it has been shown that a patient-centered integrated healthcare provides by itself an improvement of quality of life^38^.

A central role of serotonergic deficit in the ventral striatum and in the anterior cingulate cortex has recently been demonstrated in a subset of de novo PD patients exhibiting apathy, while the dopaminergic system seemed to play a less important role^15^. Thus, at disease onset, the lack of significant effect of rotigotine on apathy in PD could also be explained by the prominent role of serotonergic degeneration on these neuropsychiatric manifestation. Future controlled pharmacological trials using SSRI in de novo PD are needed in order to confirm the serotonergic involvement in apathy. Until now treatment targeting the serotonergic pathway does not seem as much effective as dopaminergic treatments in PD patients with apathy^2,14,15^. The cholinergic system is implicated in apathy, as pointed out by the positive effect of an inhibitor of acetylcholinesterase inhibitors on apathy in PD patients^14,15^. Its role in untreated PD patients should be further explored.

The number of drop offs of the study reduced the power of the study, since patients were analyzed on an intention to treat basis.

Another limitation of the study is the lack of a structured psychiatric interview in order to make the diagnosis of depression and anxiety.

The present study did not prove the efficacy of rotigotine on apathy in de novo PD patients, but a benefit on anxiety trait. There was a major placebo effect on hypodopaminergic symptoms with a biology related to mesolimbic dopaminergic denervation, but not on motor symptoms more strongly related to nigrostriatal denervation, arguing in favor of a mesolimbic origin of placebo effect. While the placebo effect may have masked potential benefit of rotigotine on apathy, overall secondary and exploratory outcomes argue in favor of a benefit of both rotigotine and of the tight follow-up of a multidisciplinary care on hypodopaminergic symptoms in de novo PD.

## Methods

This is a multicenter, prospective, parallel, randomized, controlled, double-blind trial assessing the effect of low dose rotigotine versus placebo on apathy, depression and anxiety in de novo apathetic PD patients.

### Participants

Patients were included in 6 University hospitals in France. Patients were recruited from a larger observational study on neuropsychiatric symptoms in de novo PD. Inclusion criteria were: age between 30 and 72 years, diagnosis of PD for <2 years, no cognitive impairment (defined as a score on the MATTIS Dementia rating scale <130/144^39^ or on Frontal assessment battery (FAB)^40^ <15/18), no dopaminergic treatment, no active comorbidity of major psychiatric disease (no suicidal risk, no major depressive episode according to DSM IV, no active psychosis). While patients on L-dopa or dopamine agonists were excluded, patients under rasagiline or antidepressant could be included provided that the treatment was stable within the last 3 months and remained unchanged for the rest of the study. Subjects not proficient in French language, unable to give their informed consent, pregnant or breastfeeding women were excluded from the study. All patients gave their informed written consent.

### Assessment

Patients were assessed on the day of inclusion with a detailed neurological and neuropsychological assessment including: the MDS-UPDRS for motor and non-motor symptoms severity^41^, the LARS^33^, the Starkstein apathy scale for apathy^34^, the Beck depression inventory-2 (BDI-2)^42^ for depression, the State-Trait Anxiety Inventory for anxiety trait (STAI-trait) and state (STAI-state)^43^; the Ardouin Scale of Behavior in Parkinson’s Disease for apathy, anxiety, depression and hyperdopaminergic behaviors^35^, the PDQ-39 for quality of life^44^, the MATTIS Dementia rating scale^39^ and the FAB^40^ for cognition. All the scales have been validated in PD population. Only patients with apathy, defined by a LARS score ≥ −21 were included in the randomized-controlled double-blind study.

### Study design and assessment

Apathetic patients were allocated to active treatment (rotigotine) versus placebo with a randomized ratio of 1:1. Treatment was started at 2 mg a day, and slowly titrated by step of 2 mg a week within the first 4 weeks, up to a maximal dose of 8 mg/day (recommendation for rotigotine dose range for early PD is 2–8 mg/day, in advanced PD is 4–16 mg/day). Domperidone was allowed for the management of nausea. Treatment was maintained until the end of the study, 6 months after inclusion (26 weeks). At the end of the study, patients underwent the same assessment performed at baseline. BDI-2 and Starkstein apathy scale were also performed at an intermediate visit at 3 months.

### Outcomes of the study

The primary outcome was the 6-month change in apathy, measured with the LARS score, compared to baseline. We expected a decrease in apathy, measured by a greater reduction (at least 6 points higher) in the LARS scale, in the rotigotine group compared to the placebo group.

The 6-month change in depression, measured with the BDI-2, and in anxiety, measured with the STAI-trait and STAI-state, were the secondary outcomes. We also analyzed the change at 6 months in the Starkstein apathy scale, in the items of Ardouin Scale of Behavior in PD assessing depression, anxiety, apathy and hyperdopaminergic behaviors (total score calculated as the sum of single items of hyperdopaminergic behaviors), in motor performance measured with the MDS-UPDRS III, and in quality of life measured with the PDQ-39.

### Randomization

The randomization process was centralized and generated by an external company. Randomization was stratified according to the depression score at baseline (BDI < or ≥20). Clinicians, patients, and monitors were blinded to treatment allocation. Rotigotine and placebo patches were prepared by UCB pharma and were indistinguishable.

### Sample size

We wanted to highlight a difference of at least 6 points between the two groups in the 6-month change of the LARS scale. The choice of this difference was based on the validation study of the LARS, showing a test-retest correlation coefficient of 0.95 and a classification in a different group of severity with a score difference of 6 points^33^. Assuming an inter-subject variability of 7 (standard deviation based on the results of LARS), it was necessary to include 22 subjects per group to demonstrate a difference in the LARS of at least 6 points using a two-sided test, given a power of 80% and an alpha threshold of 5%. The effectiveness highlighted corresponded to an “effect size” of 0.9, considered as important.

### Statistical analysis

The analysis was conducted using the Intention to treat analysis (ITT) population.

Data are summarized in terms of size and frequency for categorical parameters and by mean and standard deviation for continuous parameters.

The main objective analysis is based on the comparison between groups of the change of the LARS scale between baseline and 6 months (or at premature discontinuation), using multivariate analysis-of-variance (MANOVA) with repeated measures appearing as dependent variables. We tested for statistical significance a first-order interaction term involving treatment group and time.

In the case of missing data, the last observation carried forward (LOCF) method was implemented to replace the 6-month data by its value at the premature exit visit or if not available at 3 months or inclusion if available. Exploratory continuous outcomes were analyzed using the same principles. *P*-values < 0.05 were considered statistically significant.

Statistical analysis was performed using STATA release 14.2 (StataCorp, College Station, TX) PC-Software.

The trial protocol was approved by the Ethical Committee of Grenoble, authorized by the National Agency for the Safety of Medicines and Health Products (AFSSAPS) and registered as NCT02786667.

### Reporting summary

Further information on research design is available in the Nature Research Reporting Summary linked to this article.

## Supplementary information

Consortium full list

Reporting Summary Checklist

## Data Availability

Anonymized data of this study will be available from the corresponding author on reasonable request from any qualified researcher, following the EU General Data Protection Regulation. Study protocol and statistical analysis plan will be shared upon request.
