# Supplementary material for: A randomized controlled double-blind study of rotigotine on neuropsychiatric symptoms in de novo PD
Source: NPJ Parkinsons Dis. 2020 Dec 15;6:41. doi: 10.1038/s41531-020-00142-x (PMC7738499; doi:10.1038/s41531-020-00142-x)
Supplement: Supplementary file 1 — Consortium full list [file 41531_2020_142_MOESM1_ESM.pdf]

Honeymoon study group

Anna Castrioto,[1,2] Valérie Fraix,[1,2]Elena Moro,[1,2] Amélie Bichon,[1,2] Eugénie Lhommée, [1,2] Emmanuelle Schmitt,[1,2] Emmanuel Broussolle, [3,4] Stephane Thobois, [3,4] Teodor Danaila, [3,4] Chloe Laurencin, [3,4] Franck Durif, [9] Bérengère Debilly, [9] Philippe Pierre Derost, [9] Ana Marques, [9] Miguel Ulla, [9] Isabelle Chéreau-Boudet, [9] Tiphaine Vidal, [9] Jean-Luc Houeto, [11] Isabelle Benatru, [11] Solène Ansquer, [11] Jean-Philippe Azulay,[10] Tatiana Witjas, [10] Frédérique Fluchere, [10] Alexandre Eusebio, [10] Marie Delfini,[10] Christine Tranchant, [5,6,7] Mathieu Anheim, [5,6,7] Ouhaid Lagha-Boukbiza, [5,6,7] Nadine Longato, [5,6,7] Clélie Philipps, [5,6,7] Paul Krack.[13]
