# Supplementary material for: A randomized controlled double-blind study of rotigotine on neuropsychiatric symptoms in de novo PD
Source: NPJ Parkinsons Dis. 2020 Dec 15;6:41. doi: 10.1038/s41531-020-00142-x (PMC7738499; doi:10.1038/s41531-020-00142-x)
Supplement: Supplementary file 2 — Reporting Summary Checklist [file 41531_2020_142_MOESM2_ESM.pdf]

nature research

Corresponding author(s): NPJPARKD-00472

Last updated by author(s): Sep 18, 2020

Reporting Summary

Nature Research wishes to improve the reproducibility of the work that we publish. This form provides structure for consistency and transparency in reporting. For further information on Nature Research policies, see our Editorial Policies and the Editorial Policy Checklist.

Statistics

For all statistical analyses, confirm that the following items are present in the figure legend, table legend, main text, or Methods section.

n/a	Confirmed	
☐	☒	The exact sample size (*n*) for each experimental group/condition, given as a discrete number and unit of measurement
☐	☒	A statement on whether measurements were taken from distinct samples or whether the same sample was measured repeatedly
☐	☒	The statistical test(s) used AND whether they are one- or two-sided<br>*Only common tests should be described solely by name; describe more complex techniques in the Methods section.*
☐	☒	A description of all covariates tested
☐	☒	A description of any assumptions or corrections, such as tests of normality and adjustment for multiple comparisons
☐	☒	A full description of the statistical parameters including central tendency (e.g. means) or other basic estimates (e.g. regression coefficient) AND variation (e.g. standard deviation) or associated estimates of uncertainty (e.g. confidence intervals)
☐	☒	For null hypothesis testing, the test statistic (e.g. *F*, *t*, *r*) with confidence intervals, effect sizes, degrees of freedom and *P* value noted<br>*Give P values as exact values whenever suitable.*
☒	☐	For Bayesian analysis, information on the choice of priors and Markov chain Monte Carlo settings
☒	☐	For hierarchical and complex designs, identification of the appropriate level for tests and full reporting of outcomes
☒	☐	Estimates of effect sizes (e.g. Cohen's *d*, Pearson's *r*), indicating how they were calculated

Our web collection on statistics for biologists contains articles on many of the points above.

Software and code

Policy information about availability of computer code

Data collection	NA
Data analysis	Statistical analysis was performed using STATA release 14.2 (StataCorp, College Station, TX) PC-Software.

For manuscripts utilizing custom algorithms or software that are central to the research but not yet described in published literature, software must be made available to editors and reviewers. We strongly encourage code deposition in a community repository (e.g. GitHub). See the Nature Research guidelines for submitting code & software for further information.

Data

Policy information about availability of data

All manuscripts must include a data availability statement. This statement should provide the following information, where applicable:

- Accession codes, unique identifiers, or web links for publicly available datasets
- A list of figures that have associated raw data
- A description of any restrictions on data availability

Anonymized data of this study will be available from the corresponding author on reasonable request from any qualified researcher, following the EU General Data Protectioin Regulation. Study protocol and statistical analysis plan will be shared upon request.

Field-specific reporting

Please select the one below that is the best fit for your research. If you are not sure, read the appropriate sections before making your selection.

☒ Life sciences ☐ Behavioural & social sciences ☐ Ecological, evolutionary & environmental sciences

For a reference copy of the document with all sections, see nature.com/documents/nr-reporting-summary-flat.pdf

Life sciences study design

All studies must disclose on these points even when the disclosure is negative.

Sample size	We wanted to highlight a difference of at least 6 points between the 2 groups in the 6-month change of the LARS scale. The choice of this difference was based on the validation study of the LARS, showing a test-retest correlation coefficient of 0.95 and a classification in a different group of severity with a score difference of 6 points.33 Assuming an inter-subject variability of 7 (standard deviation based on the results of LARS), it was necessary to include 22 subjects per group to demonstrate a difference in the LARS of at least 6 points using a two-sided test, given a power of 80% and an alpha threshold of 5%. The effectiveness highlighted corresponded to an "effect size" of 0.9, considered as important.
Data exclusions	N/A. ITT Analysis: no data were excluded.
Replication	Using two statistical analysis approaches (ANCOVA and MANOVA) reproduced the same results.
Randomization	The randomization process was centralized and generated by an external company. Randomization was stratified according to the depression score at baseline (BDI< or > 20).
Blinding	Clinicians, patients, and monitors were blinded to treatment allocation. Rotigotine and placebo patches were prepared by UCB pharma and were indistinguishable.

Reporting for specific materials, systems and methods

We require information from authors about some types of materials, experimental systems and methods used in many studies. Here, indicate whether each material, system or method listed is relevant to your study. If you are not sure if a list item applies to your research, read the appropriate section before selecting a response.

Materials & experimental systems

n/a	Involved in the study	
☒	☐	Antibodies
☒	☐	Eukaryotic cell lines
☒	☐	Palaeontology and archaeology
☒	☐	Animals and other organisms
☐	☒	Human research participants
☐	☒	Clinical data
☒	☐	Dual use research of concern

Methods

n/a	Involved in the study	
☒	☐	ChIP-seq
☒	☐	Flow cytometry
☒	☐	MRI-based neuroimaging

Human research participants

Policy information about studies involving human research participants

Population characteristics	Patients were recruited from a larger observational study on neuropsychiatric symptoms in de novo PD. Inclusion criteria were: age between 30 and 72 years, diagnosis of PD for less than 2 years, no cognitive impairment (defined as a score on the MATTIS Dementia rating scale <130/14439 or on Frontal assessment battery40 <15/18), no dopaminergic treatment, no active comorbidity of major psychiatric disease (no suicidal risk, no major depressive episode according to DSM IV, no active psychosis). While patients on L-dopa or dopamine agonists were excluded, patients under rasagiline or antidepressant could be included provided that the treatment was stable within the last 3 months and remained unchanged for the rest of the study. Subjects not proficient in French language, unable to give their informed consent, pregnant or breastfeeding women were excluded from the study. All patients gave their informed written consent. Baseline characteristics of patients are reported in table 1 of the manuscript.
Recruitment	Patients were recruited from a larger observational study on neuropsychiatric issues in de novo PD
Ethics oversight	The trial protocol was approved by the Ethical Committee of Grenoble, authorized by the National Agency for the Safety of Medicines and Health Products (AFSSAPS).

Note that full information on the approval of the study protocol must also be provided in the manuscript.

Clinical data

Policy information about clinical studies

All manuscripts should comply with the ICMJE guidelines for publication of clinical research and a completed CONSORT checklist must be included with all submissions.

Clinical trial registration	NCT02786667
Study protocol	Study protocol is available upon request
Data collection	This is a multicenter study, conducted in 6 University hospitals in France. Patients were recruited from June 2012 to april 2017.
Outcomes	The primary outcome was the 6-month change in apathy, measured with the LARS score, compared to baseline.<br>The 6-month change in depression, measured with the BDI-2, and in anxiety, measured with the STAI-trait and STAI-state, were the secondary outcomes. All the outcome measures are validated in PD population.
